# Botulism in a Dog Fed a Raw Meat-Based Diet: A Case Report

**DOI:** 10.3390/microorganisms14010192

**Published:** 2026-01-15

**Authors:** Flávia Mello Viegas, Poliane de Fátima Oliveira, Marina Carvalho Oliveira Campos, Marina Mendes Santiago Fernandes, Alexandra Oliveira Abreu, Clara Berquo Cascaes, João Victor Ferreira Campos, Rodrigo Otávio Silveira Silva

**Affiliations:** 1 Hospital Veterinário MedVet, Belo Horizonte 31720200, Brazil; 2 Departamento de Medicina Veterinária Preventiva, Universidade Federal de Minas Gerais (UFMG), Belo Horizonte 30720440, Brazil

**Keywords:** public health, RMBD, botulinic toxin, paralysis

## Abstract

Raw meat-based diets (RMBDs) have become increasingly popular among pet owners, despite well-documented risks of contamination with pathogenic bacteria capable of causing severe illness in companion animals. This report describes a fatal case of botulism in a 3-year-old female Labrador Retriever weighing 37 kg that was fed exclusively RMBD. The dog presented with acute-onset flaccid paralysis of the limbs approximately 48 h after possible ingestion of decomposing raw meat discarded in household waste. Supportive therapy, including fluid administration, nutritional support and eventual mechanical ventilation was provided. However, the patient developed progressive respiratory failure and died. The presence of *Clostridium botulinum* type C neurotoxin was confirmed in the dog serum by neutralization test in mice. The case suggests RMBD as a potential source of botulism toxins, particularly when derived from improperly stored meat products. The findings underscore the importance of detailed dietary history in dogs presenting with acute flaccid paralysis and reinforce the need for heightened awareness regarding the microbiological risks associated with raw feeding practices.

## 1. Introduction

In recent years, unconventional feeding practices for dogs and cats, particularly raw meat-based diets (RMBDs), have gained popularity among pet owners seeking to provide diets perceived as more “natural” and “healthier” [1,2,3,4]. While commercial dry and wet foods continue to represent the primary nutrition source for most companion animals, evidence suggests that nearly half of all dogs currently receive at least one type of raw meat product as part of their regular feeding regimen, mostly in European and North American countries [4,5,6,7,8].

Despite the purported benefits, RMBDs are frequently associated with contamination by pathogenic bacteria, including *Salmonella* spp., *Escherichia coli*, *Campylobacter* spp., and *Clostridium* spp., which has been linked to an increased fecal shedding of those pathogens, thereby creating potential health risks for both pets and humans [1,9,10,11]. Moreover, there are reports of fatal outcomes in animals exposed to raw diets contaminated with pathogens causing tuberculosis, brucellosis, and avian influenza [7,12,13,14].

Particularly concerning are spore-forming bacteria, such as *Clostridium* spp., which can survive under conditions that would typically inactivate vegetative cells, including low-temperature storage and minimal thermal processing [15]. These organisms pose a heightened risk for severe intoxications, including botulism, a rare but potentially fatal condition in dogs [16]. This report aims to describe a case of botulism in a dog fed exclusively on a raw meat-based diet.

## 2. Case Report

A 3-year-old female Labrador Retriever, weighing 37 kg, with a body condition score of 6/9 and a muscle mass score of 3/3, was presented to a private veterinary clinic in Belo Horizonte, Minas Gerais State, Brazil, with a history of acute-onset paraplegia. The patient had no previous medical conditions, was fully vaccinated (Nobivac^®^ DHPPI+L and Nobivac^®^ Rabies, MSD, USA), and lived in an apartment with two other female dogs. According to the owner, the dog had no access to outdoor or rural environments and was exclusively fed a RMBD prescribed by a veterinarian. The diet was home-prepared weekly and kept frozen. All dogs were fed twice daily and individual portions were thawed the day before being offered. The diet consisted primarily of meaty poultry bones (35%), poultry breast (35%), cattle liver (5%), and vegetables (25%), totaling approximately 900 g per day.

On physical examination, the patient exhibited flaccid tetraparesis with preserved consciousness and intact superficial and deep nociception. Proprioceptive positioning could not be assessed due to the degree of motor impairment. Based on the clinical presentation, the main differential diagnoses included botulism, myasthenia gravis, and acute polyradiculoneuritis. Upon further questioning, the owner reported a possible episode of ingestion of raw meat scraps discarded in household waste approximately two days before the onset of neurological signs.

The patient was hospitalized and received supportive therapy, including intravenous fluid administration, prophylactic antimicrobial therapy (Metronidazole 15 mg/kg twice a day), prokinetic agents (metoclopramide 0.5 mg/kg three times a day), and antiemetic medication (ondansetron 0.5 mg/kg three times a day). Enteral nutritional support was initiated via a nasogastric feeding tube. A commercial dry diet formulated for gastrointestinal disorders was used, and the daily caloric intake was determined using the resting energy requirement (RER) equation. A urinary catheter was also placed to facilitate bladder emptying, and enemas were performed for fecal evacuation, during which a considerable quantity of bone fragments was observed in the stool.

Complete blood count and serum biochemical analysis revealed no significant abnormalities, except for mild hypertriglyceridemia and hypercholesterolemia. Abdominal ultrasonography demonstrated mild gastric distension, a small amount of fecal material within the intestinal tract, and ultrasonographic evidence suggestive of acute hepatopathy, including mild hepatomegaly, mildly hyperechoic liver and slightly heterogeneous echotexture.

A serum sample was collected on the first day of admission and submitted to the Laboratory of Anaerobes, Department of Preventive Veterinary Medicine, Federal University of Minas Gerais (UFMG). The sample was centrifuged at 10,000 × *g* for 10 min at 4 °C and subsequently subjected to the mouse serum neutralization test, which is considered the gold standard for the diagnosis of botulism [17]. Briefly, serum dilutions (1:10 and 1:100) were intraperitoneally inoculated into mice, which were then observed for 48 h. In the present case, all inoculated mice died within 24 h. To confirm the presence of botulinum neurotoxin and identify the toxin serotype, the serum sample was mixed with *C. botulinum* type C antitoxin (NIBSC, UK) prior to inoculation (mouse neutralization test). All mice survived following antitoxin neutralization, confirming the diagnosis of type C botulism.

After three days of hospitalization, the patient developed multiple episodes of vomiting and exhibited clinical signs consistent with laryngeal paralysis. Due to progressive respiratory compromise, the dog was transferred to an intensive care unit to receive mechanical ventilation therapy. Despite intensive supportive management, the patient experienced cardiorespiratory arrest and died four days after the onset of clinical signs.

## 3. Discussion

Botulism is an uncommon but potentially fatal neuroparalytic disorder affecting a wide range of mammals and birds [16,18]. The clinical signs observed in the dog described in this report are consistent with those commonly reported in other cases, including initial limb weakness that rapidly progresses to the characteristic flaccid paralysis with preserved mental status. Vomiting, dehydration, inappetence, and fecal and urinary retention may also be observed [16,18,19,20].

In canine patients, diagnosis is frequently presumptive and based primarily on clinical presentation, as confirmatory laboratory testing can be difficult to perform [19]. In the current report, however, laboratory analysis successfully identified *C. botulinum* type C toxin in the dog’s serum, thus confirming the diagnosis. Interestingly, *C. botulinum* type C is commonly implicated in animal botulism [21,22] including canine botulism [16,20,23].

Noteworthy, canine botulism is most often associated with the ingestion of decomposing animal carcasses or spoiled food waste, with the former representing the predominant source of exposure in Brazil [20,24,25]. Clinical signs generally develop within 12 to 24 h after toxin ingestion, although in some instances, the incubation period may extend up to five days [20,21]. In the present case, neurological signs were first observed approximately 48 h after the suspected exposure. The owners reported the possibility that the dog had ingested decomposing raw meat discarded in household waste, although this could not be conclusively verified.

Unfortunately, it was not possible to perform microbiological testing in the suspected source of toxin. Once the neutralization test is known to have a low sensitivity [26] it would be interesting to quantify de presence of *C. botulinum* type C spores by molecular methods in the suspected feed, which would help to elucidate the likely source of toxin. Anyway, despite it not being possible to identify the *C. botulinum* neurotoxin in food sample, given that the dog was maintained exclusively on a RMBD, it is plausible that the contamination originated from the same food products that were discarded and subsequently accessed by the animal.

*C. botulinum* is a spore-forming bacterium commonly present in soil and in the gastrointestinal tract of food-producing animals, particularly poultry [27]. Consequently, raw meat and by-products derived from this specie may become contaminated during slaughter or handling, allowing spores to persist in the final product and pose a potential source of intoxication when consumed without prior cooking or sterilization [28]. In RMBDs, the absence of thermal processing permits spore survival, and inadequate refrigeration or prolonged storage may promote toxin formation [15]. These factors underscore the potential hazards associated with raw feeding practices, especially when proper hygiene and cold-chain control are not strictly maintained.

Although the source of the toxin was not confirmed, this report serves as an alert regarding proper food handling and disposal, particularly when RMBDs are used. It is also important to note that strong scientific evidence demonstrates that RMBDs can serve as a relevant source of antimicrobial resistant determinants and zoonotic pathogens, posing risks not only to pets but also to humans in close contact with them [2,4,7,11,29,30]. Thus, the RMBD has been discouraged by several organizations, including the World Small Animal Veterinary Association (WSAVA), the Centers for Disease Control and Prevention (CDC), and the Food and Drug Administration (FDA) [29,31,32].

## 4. Conclusions

The present report underscores the need for veterinarians to consider botulism as a differential diagnosis in dogs presenting with acute flaccid paralysis and maintained on a RMBD. Early recognition of clinical signs, combined with a thorough dietary history, is essential for timely intervention. Specific antidotes for botulinum toxin are not currently available for dogs in most countries; thus, supportive care remains the cornerstone of treatment.

## Data Availability

The original contributions presented in this study are included in the article. Further inquiries can be directed to the corresponding author.
